# Three new species (*Coniochaetales*, *Eremomycetales*, *Spiromycetales*) isolated from rhizosphere soil of tea plant (*Camellia sinensis*) in Guizhou, China

**DOI:** 10.3897/mycokeys.134.186271

**Published:** 2026-06-10

**Authors:** Hai-Yan Wang, Hong-Qin Qu, Yu-Feng Zhao, Ke-Yun Song, Hai-Long Zhang, Yan-Ling Wang, Chun-Bo Dong, Yan-Wei Zhang, Wan-Hao Chen, Yan-Feng Han

**Affiliations:** 1 Institute of Fungus Resources, Department of Ecology/ Guizhou Key laboratory of Agricultural Microbiology, College of Life Science, Guizhou University, Guiyang, 550025, Guizhou, China Key Laboratory of Development and Utilization of Biological Resources in Colleges and Universities of Guizhou Province/ Key Laboratory of Ecology and Management on Forest Fire in Higher Education institutions of Guizhou Province, Guizhou Education University Guiyang China; 2 Key Laboratory of Development and Utilization of Biological Resources in Colleges and Universities of Guizhou Province/ Key Laboratory of Ecology and Management on Forest Fire in Higher Education institutions of Guizhou Province, Guizhou Education University, Guiyang, 550018, Guizhou, China Institute of Fungus Resources, Department of Ecology/ Guizhou Key laboratory of Agricultural Microbiology, College of Life Science, Guizhou University Guiyang China; 3 Center for Mycomedicine Research, Basic Medical School, Guizhou University of Traditional Chinese Medicine, Guiyang, 550025, Guizhou, China Center for Mycomedicine Research, Basic Medical School, Guizhou University of Traditional Chinese Medicine Guiyang China

**Keywords:** *
Coniochaetaceae
*, *
Eremomycetaceae
*, fungal taxonomy, multi-gene phylogeny, new taxa, rhizosphere fungi, *
Spiromycetales
*

## Abstract

Rhizosphere soil, serving as the micro-ecological interface linking plant roots and the surrounding environment, is critical for the tea plant, as it harbors a rich diversity of microbial species that influence nutrient absorption and transformation in plant roots. During a fungal diversity survey of rhizosphere soil from tea plants, 10 fungal strains were isolated from soil samples collected in Guizhou, China. Based on morphological characteristics and multi-gene phylogenetic analysis, they were identified and proposed as three new species: *Arthrographis guizhouensis* sp. nov., *Coniochaeta guizhouensis* sp. nov., and *Spiromyces sinensis* sp. nov. In this study, morphological descriptions, illustrations, and molecular phylogenetic analyses of these three new species are presented.

## Introduction

Tea [*Camellia sinensis* (L.) O. Kuntze] is an economically important perennial nonalcoholic beverage crop cultivated in most provinces of China (Tang et al. 2021) and is widely grown in different countries of Africa and Asia (Bhattacharyya et al. 2022). According to the 2024 report on the production and marketing situation of tea in China, the tea plant planting area was 3,495,220 hectares, the total output of tea was 3.4991 million tons, and the total output value of tea was 321.785 billion yuan in China. Tea farmers and researchers have long made considerable efforts to ensure the production of high-quality tea. High-quality tea depends on healthy tea trees. A healthy soil environment provides a strong guarantee for the health of tea plants and the quality of fresh tea (Xie et al. 2025). However, soil microorganisms play a crucial role in the growth of healthy tea plants. The microbial community structure of the rhizosphere is an important indicator of soil quality in tea gardens (Wang 2022).

Fungi are among the most diverse organisms on Earth. They are widely distributed across various environments and play important roles in ecosystem processes and functions (Zhang et al. 2023b; Zhang et al. 2024; Wang et al. 2025). Soil fungi, in particular, regulate key ecological functions, including soil fertility improvement, mineral decomposition, and organic matter cycling, while also affecting plant health and nutrient acquisition (Wei et al. 2023; Wang et al. 2025). The rhizosphere, a micro-environment strongly influenced by plant roots, serves as a critical interface between plants and soil (Ding et al. 2022). It constitutes a distinct micro-ecosystem (Zhang et al. 2023b). The structure, composition, and diversity of fungal communities in rhizosphere soil are shaped by plant roots through the regulation of the types and quantity of root exudates (Ding et al. 2022; He et al. 2025). Conversely, rhizosphere soil fungi affect root exudates by altering nutrient transformation and uptake in the rhizosphere, thereby affecting plant growth, metabolism, and development (Li et al. 2021; Zhang et al. 2023a). Some of these rhizosphere fungi were considered closely related to the health, metabolism, and growth of tea plants (Zhu et al. 2024). However, relatively few studies have focused on culturable fungal diversity in the rhizosphere soil of tea plants; most have relied on high-throughput sequencing data, with limited attention given to culturable taxa (Zhang et al. 2018; Wei et al. 2023; Zhu et al. 2024). Therefore, greater attention should be paid to the composition, diversity, and function of culturable fungi in the rhizosphere soil of tea plants.

To date, various fungi have been reported from the roots, stems, leaves, and rhizosphere soil of tea plants in China, including culturable and unculturable taxa (Win et al. 2018; Tibpromma et al. 2022; Yan et al. 2024; Zhu et al. 2024). Nevertheless, most of these discoveries, particularly those based on high-throughput sequencing, have focused on unculturable communities. While such approaches provide valuable insights into overall fungal diversity, they do not yield culturable strains for further morphological and functional studies, which are important for fungal taxonomy. For instance, a wide array of fungi has been discovered from the rhizosphere soil of tea plants (Zhu et al. 2024). Wang et al. (2024) reported *Basidiomycota*, *Mortierellomycota*, and *Ascomycota* as the dominant phyla in tea rhizosphere soil, with their distributions correlated with pH, total phosphorus, exchangeable magnesium, exchangeable calcium, available phosphorus, and available potassium. Total nitrogen, total phosphorus, available phosphorus, organic matter, and water-soluble nitrogen are the environmental drivers of variation in fungal β-diversity. Similarly, Zhao et al. (2019) reported *Basidiomycota*, *Ascomycota*, and *Zygomycota* as dominant fungal groups, with *Cryptococcus* spp. being the most abundant fungi across four tea plant cultivars. Yan et al. (2024) documented fungal genera such as *Aspergillus* P. Micheli ex Haller, *Cladophialophora* Borelli, *Endocarpon* Hedw., *Exophiala* J.W. Carmich., *Fusarium* Link, *Rhizophagus* P.A. Dang., *Rhizopus* Ehrenb., *Saitozyma* Xin Zhan Liu, F.Y. Bai, M. Groenew. & Boekhout, *Suillus* Gray, and *Tricholoma* (Fr.) Staude in tea rhizosphere soils under varying environmental conditions. The fungal community in tea rhizosphere soil is co-regulated by root secretions, growth conditions, fertilization practices, and temperature stress (Feng et al. 2024). However, reports on culturable fungi from the rhizosphere soil of tea plants remain limited. In Guizhou Province, a major tea-growing province in China, the province ranks first in both planting area and output value (Huang 2023). Research on rhizosphere soil fungi of tea plants in Guizhou Province is scattered and thus requires systematic evaluation.

Based on the above background, an exploratory survey of culturable fungi in the rhizosphere soil of tea plants (*Camellia sinensis*) was conducted in Guizhou, China. Ten fungal strains were isolated, purified, identified, and proposed as three new taxa, including *Arthrographis guizhouensis* sp. nov., *Coniochaeta guizhouensis* sp. nov., and *Spiromyces sinensis* sp. nov., based on multi-gene (ITS, LSU, SSU, and ACT) phylogenetic analysis and morphological characteristics. Detailed descriptions and illustrations of three new taxa are provided in this study.

## Materials and methods

### Sample collection and fungal isolation

The bulk soil was gently shaken off, and the rhizosphere soil adhering to the tea root surface (approximately 1–2 mm) was collected into sterile sealed bags using a small brush (Ran et al. 2024). Soil samples were placed in Ziploc plastic bags and brought back to the laboratory. For fungal isolation, 2 g of each soil sample was placed into a sterile 50 mL conical flask containing 20 mL sterile water and thoroughly shaken using a vortex vibration meter. The soil samples were diluted to a suspension with a concentration of 10^−3^ spores/mL. Then, 1 mL of the diluted sample was transferred to a sterile Petri dish with three different modified media: Sabouraud’s dextrose agar (SDA, peptone 10 g/L, dextrose 40 g/L, agar 20 g/L, 3.3 mL of 1% Bengal red aqueous solution), Martin’s medium (KH2PO4 1 g/L, MgSO4 0.5 g/L, peptone 5 g/L, glucose 2 g/L, agar 20 g/L, 1% Bengal red aqueous solution 3.3 mL/L), and Sabouraud’s dextrose agar yeast extract (SDAY, peptone 10 g/L, dextrose 40 g/L, agar 20 g/L, yeast extract 2 g/L, 1% Bengal red aqueous solution 3.3 mL/L), containing 50 mg/L penicillin and 50 mg/L streptomycin to inhibit bacterial growth. The plates were incubated at 25 °C for 7 d, and then each single colony was selected from the plates and transferred to new potato dextrose agar (PDA, potato 200 g/L, dextrose 20 g/L, agar 20 g/L) plates.

### Morphological study

Fungal cultures were cultivated on fresh malt extract agar (MEA, malt extract 30 g/L, agar 20 g/L, peptone 10 g/L), oatmeal agar (OA, oatmeal 30 g/L, agar 20 g/L), and potato dextrose agar (PDA, potato 200 g/L, dextrose 20 g/L, agar 20 g/L) and were incubated at 25 °C to examine their colony morphology and microscopic morphology. After 14 d, colony colors (according to the national standard color card) and diameters were observed and recorded. Meanwhile, fungal reproductive structures were examined on PDA and captured by making direct wet mounts with 25% lactic acid using an optical microscope (BX53, OLYMPUS). Strains generated from this study were deposited in the Institute of Fungus Resources, Guizhou University (GZUIFR = GZAC). Taxonomic descriptions and nomenclature of three new species were uploaded to MycoBank (https://www.mycobank.org/, accessed on 23 January 2026).

### DNA extraction, PCR amplification, and sequencing

Mycelium (0.1 mg) was collected and added to a centrifuge tube with 100 μL of 5% Chelex-100 solution, bathed in water at 100 °C for 10 min, and centrifuged at 12,000 rpm for 1 min. Finally, the liquid supernatant was obtained as total genomic DNA. The extracted DNA was stored at −20 °C. Primer pairs ITS1/ITS4 (White et al. 1990), LR0R/LR5 (Vilgalys and Hester 1990), NS1/NS4 (White et al. 1990), and ACT1/ACT4 (Voigt and Wöstemeyer 2000) were used for PCR amplification of the internal transcribed spacers (ITS), the 28S nrRNA locus (LSU), the 18S small subunit nuclear rRNA gene (SSU), and the actin gene (ACT), respectively. The PCR amplification conditions for ITS were as follows: initial denaturation at 94 °C for 5 min, followed by 35 cycles of denaturation at 94 °C for 30 s, annealing at 51 °C for 50 s, and extension at 72 °C for 45 s, with a final extension at 72 °C for 10 min. The PCR amplification conditions for LSU were as follows: initial denaturation at 94 °C for 5 min, followed by 35 cycles of denaturation at 94 °C for 30 s, annealing at 51 °C for 1 min, and extension at 72 °C for 2 min, with a final extension at 72 °C for 10 min. The PCR amplification conditions for SSU were as follows: initial denaturation at 94 °C for 3 min, followed by 40 cycles of denaturation at 94 °C for 1 min, annealing at 53 °C for 30 s, and extension at 72 °C for 1 min, with a final extension at 72 °C for 10 min. The PCR amplification conditions for ACT were as follows: initial denaturation at 94 °C for 1 min, followed by 40 cycles of denaturation at 95 °C for 1 min, annealing at 55 °C for 1 min, and extension at 72 °C for 1 min, with a final extension at 72 °C for 10 min. The PCR products were sent to Quintarabio (Wuhan, China) for purification and sequencing. In this study, strain sequences were submitted to GenBank (https://www.ncbi.nlm.nih.gov/) (Suppl. material 1: tables S1–S3).

### Phylogenetic analysis

The relevant strain sequences were downloaded from GenBank based on recent related studies (Li et al. 2022; Ri et al. 2022, Silva et al. 2023; Crous et al. 2025; Ri and Degawa 2025), and other sequences were selected based on BLASTn similarity searches (Suppl. material 1: tables S1–S3). The multiple datasets of ITS, LSU, SSU, and ACT genes were aligned and trimmed in MEGA v.6.06 (Tamura et al. 2013). Three phylogenetic analyses were performed in PhyloSuite v.1.2.2 (Zhang et al. 2020) to confirm the placement of the isolates (Analysis 1: ITS, LSU, and SSU; Analysis 2: ITS and LSU; Analysis 3: ACT, ITS, and LSU gene regions).

Using the “Concatenate Sequence” function, the concatenation of loci was conducted in PhyloSuite v.1.2.2 (Zhang et al. 2020). The phylogenetic construction of each locus dataset was processed by maximum likelihood (ML) and Bayesian inference (BI) methods. In ModelFinder, the Akaike Information Criterion correction (AICc) was used for the best-fit substitution model (Kalyaanamoorthy et al. 2017). With 1,000 bootstrap tests using the ultrafast algorithm (Minh et al. 2013), ML analysis was conducted using IQ-TREE (Nguyen et al. 2015) in PhyloSuite v.1.2.2. BI analysis was performed using MrBayes (Ronquist et al. 2012) in PhyloSuite v.1.2.2, and Markov chain Monte Carlo (MCMC) simulations were used for 2 × 10^6^ generations. The phylogenetic trees were visualized in FigTree version 1.4.3 and edited in Adobe Illustrator 2020 and PowerPoint 2019.

## Results

### Phylogenetic analyses

#### Analysis 1

**Figure 1.**
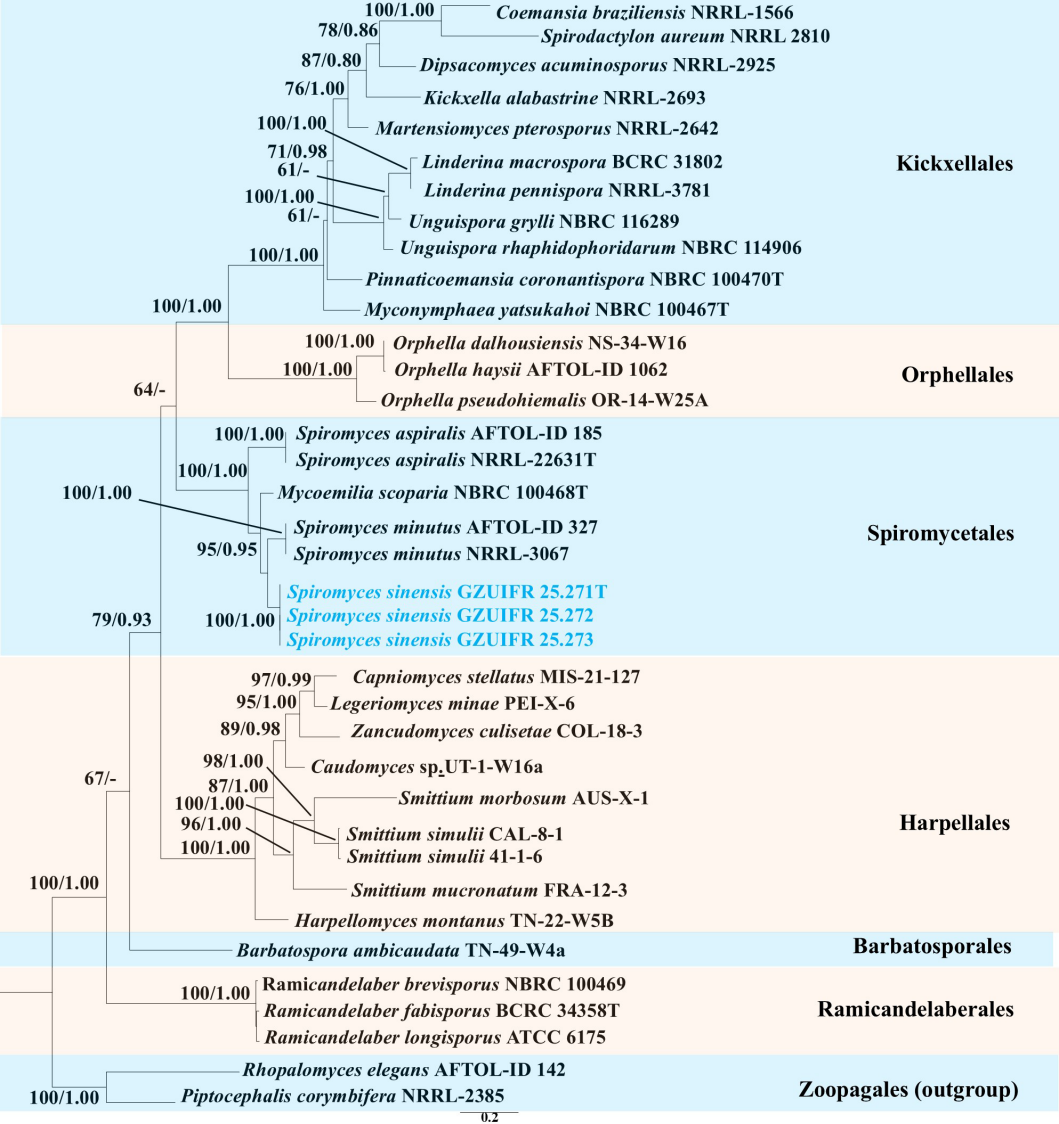
Phylogenetic tree of *Kickxellomycotina* constructed from ML analyses of a combined ITS, LSU, and SSU sequence dataset. ML bootstrap values ≥ 50% and BI posterior probabilities ≥ 0.70 are given at the nodes (MLBS/BYPP). “–” indicates the absence of statistical support. The tree is rooted to *Piptocephalis corymbifera* and *Rhopalomyces elegans*. Ex-type strains are indicated by “T” after the strain number, and the new species is indicated in blue bold.

The phylogenetic tree was constructed to determine the establishment of the new species in *Spiromycetales* (Fig. 1). The phylogenetic placement of three newly generated strains (GZUIFR 25.271, GZUIFR 25.272, and GZUIFR 25.273) was determined based on the combined ITS (1–404 bp), LSU (405–922 bp), and SSU (923–2113 bp) sequence dataset, consisting of 2113 characters, including gaps. The aligned sequence dataset comprised 37 representative taxa in *Kickxellomycotina*, with *Piptocephalis corymbifera* Vuill. and *Rhopalomyces elegans* Corda in *Zoopagales* as the outgroup taxa. The selected model for ML analysis was TIM3+F+I+G4. The final value of the best-scoring ML tree was –23259.907 and was selected to represent phylogenetic relationships among taxa in *Kickxellomycotina*. Estimated base frequencies were as follows: A = 0.275, C = 0.186, G = 0.264, T = 0.274; substitution rates AC = 0.76843, AG = 1.98905, AT = 1.00000, CG = 0.76843, CT = 3.23480, GT = 1.00000; and a gamma distribution shape parameter (α) of 0.701. For BI analysis, the best-fit model identified for the dataset was GTR+F+I+G4. The phylogenetic tree was consistent and strongly supported in branches based on ML and BI analyses. Phylogenetic analyses demonstrated that orders of *Kickxellomycotina* (viz. *Barbatosporales*, *Harpellales*, *Kickxellales*, *Orphellales*, *Ramicandelaberales*, and *Spiromycetales*) formed well-resolved clades (100% MLBS and 1.00 BYPP; Fig. 1), except for *Barbatosporales*, which formed an independent lineage basal to *Harpellales* (67% MLBS). In the *Spiromycetales* clade, three newly generated strains (GZUIFR 25.271, GZUIFR 25.272, and GZUIFR 25.273) shared the same branch length with a high support value (100% MLBS and 1.00 BYPP; Fig. 1) within *Spiromycetales*, constituting a basal lineage to *Spiromyces minutus* (AFTOL-ID 327 and NRRL-3067) with significant support (95% MLBS and 0.95 BYPP; Fig. 1). Therefore, a new species, *Spiromyces sinensis* sp. nov., is introduced herein to the genus *Spiromyces*. Notably, *Spiromyces aspiralis* (AFTOL-ID 185 and NRRL-22631) formed a separate branch, distant from *S. minutus* in the present study. The congeneric status of these two species requires further study.

#### Analysis 2

**Figure 2.**
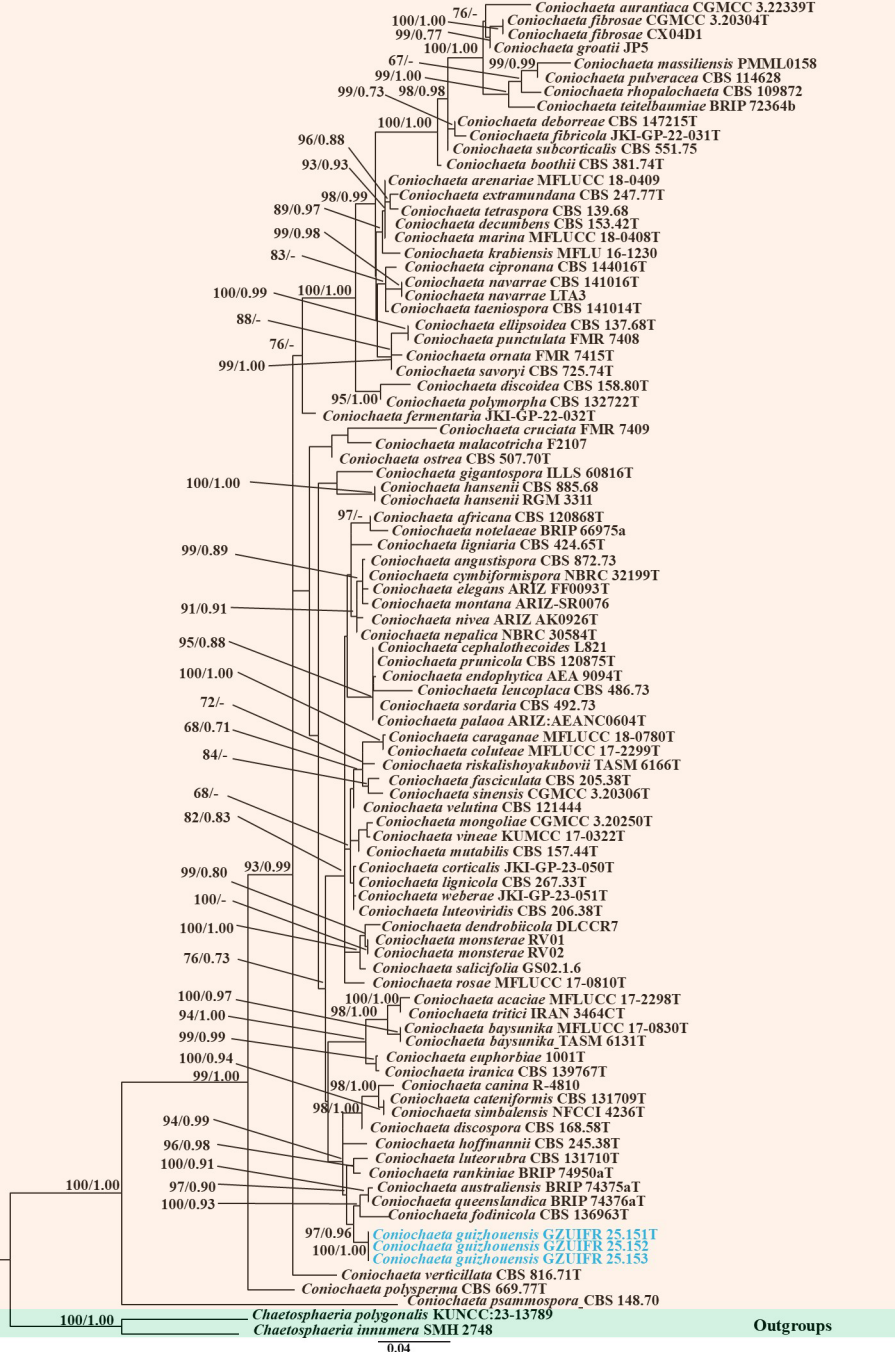
Phylogenetic tree of *Coniochaeta* constructed from ML analyses of a combined ITS and LSU sequence dataset. ML bootstrap values ≥ 50% and BI posterior probabilities ≥ 0.70 are given at the nodes (MLBS/BYPP). “–” indicates the absence of statistical support. The tree is rooted to *Chaetosphaeria innumera* and *C. polygonalis*. Ex-type strains are indicated by “T” after the strain number, and the new species is indicated in blue bold.

The phylogenetic tree was constructed to determine the establishment of the new species in *Coniochaetaceae* (Fig. 2). The phylogenetic placement of three strains (GZUIFR 25.151, GZUIFR 25.152, and GZUIFR 25.153) was determined based on the combined ITS (1–323 bp) and LSU (324–762 bp) sequence dataset, consisting of 762 characters, including gaps. The aligned sequence dataset comprised 92 representative taxa in the genus *Coniochaeta*, with *Chaetosphaeria innumera* Berk. & Broome ex Tul. & C. Tul. and *C. polygonalis* Jian Yang, Jian K. Liu & K.D. Hyde as the outgroup taxa. The selected model for ML analysis was TIM3e+R3. The final value of the best-scoring ML tree was –4694.716 and was selected to represent phylogenetic relationships among taxa in *Coniochaeta*. Estimated base frequencies were as follows: A = 0.250, C = 0.250, G = 0.250, T = 0.250; substitution rates AC = 1.45810, AG = 2.48478, AT = 1.00000, CG = 1.45810, CT = 7.38675, GT = 1.00000. For BI analysis, the best-fit model identified for the dataset was SYM+I+G4. The phylogenetic tree was consistent and strongly supported in branches based on ML and BI analyses. Phylogenetic analyses demonstrated that the genus *Coniochaeta* formed a distinct clade (97% MLBS and 0.96 BYPP) and appeared to be separated from *Coniochaeta fodinicola* Vázq.-Camp. (CBS 136963), *C. queenslandica* Y.P. Tan & Bishop-Hurley (BRIP 74376a), and *C. australiensis* Y.P. Tan & Bishop-Hurley (BRIP 74375a). The three newly generated strains (GZUIFR 25.151, GZUIFR 25.152, and GZUIFR 25.153) shared the same branch length with a high support value (100% MLBS and 1.00 BYPP; Fig. 2). Therefore, *Coniochaeta guizhouensis* sp. nov. is introduced herein to the genus *Coniochaeta*.

#### Analysis 3

**Figure 3.**
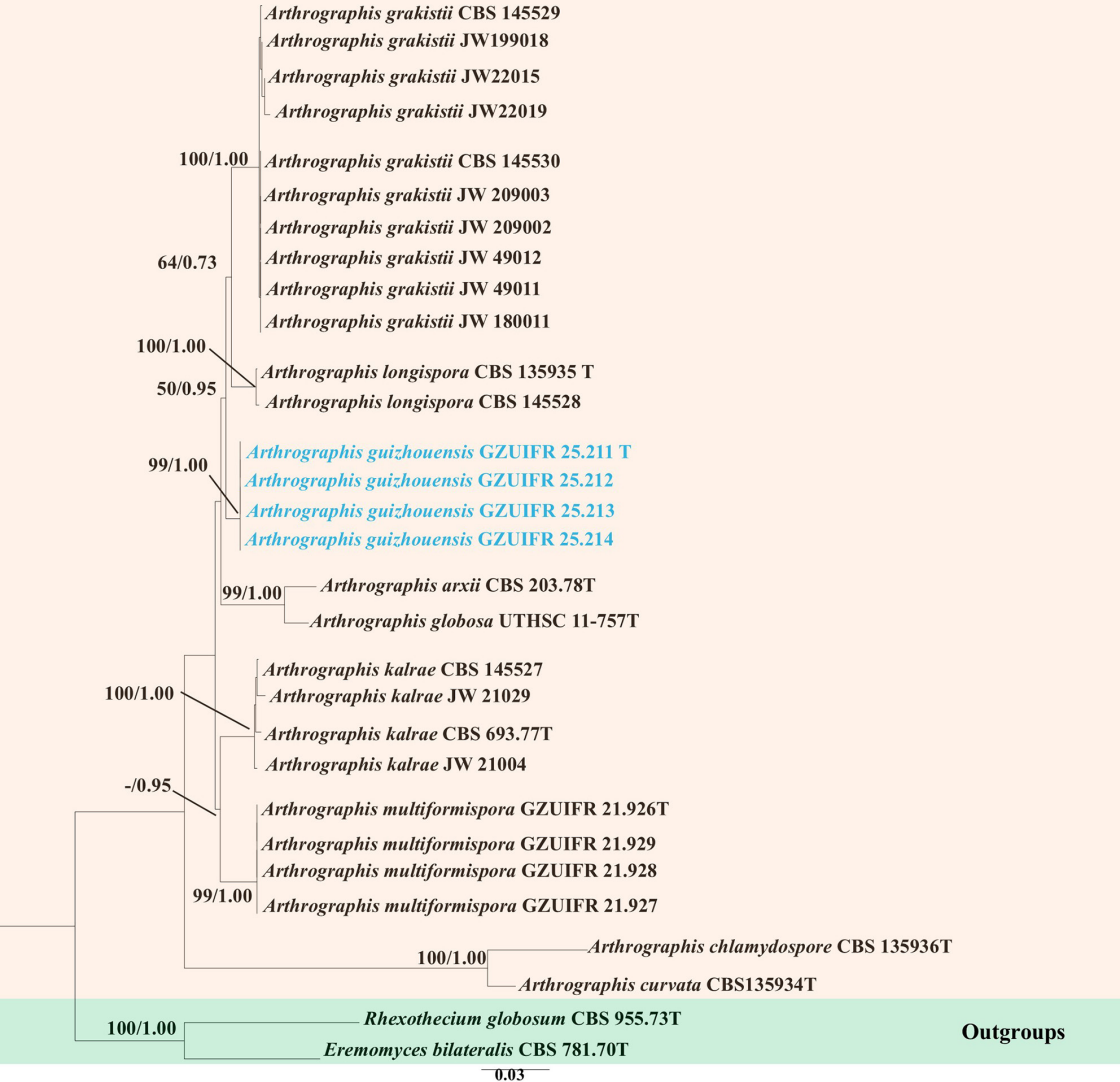
Phylogenetic tree of *Arthrographis* constructed from ML analyses of a combined ACT, ITS, and LSU sequence dataset. ML bootstrap values ≥ 50% and BI posterior probabilities ≥ 0.70 are given at the nodes (MLBS/BYPP). “–” indicates the absence of statistical support. The tree is rooted to *Eremomyces bilateralis* and *Rhexothecium globosum*. Ex-type strains are indicated by “T” after the strain number, and the new species is indicated in blue bold.

The phylogenetic tree was constructed to determine the establishment of the new species in *Arthrographis* (Fig. 3). The phylogenetic placement of four newly generated strains (GZUIFR 25.211, GZUIFR 25.212, GZUIFR 25.213, and GZUIFR 25.214) was determined based on the combined ACT (1–771 bp), ITS (772–1239 bp), and LSU (1240–1759 bp) sequence dataset, consisting of 1759 characters, including gaps. The aligned sequence dataset comprised 30 representative taxa in the genus *Arthrographis*, with *Eremomyces bilateralis* Malloch & Cain and *Rhexothecium globosum* Samson & Mouch. as the outgroup taxa. The selected model for ML analysis was GTR+F+R3. The final value of the best-scoring ML tree was –5563.139 and was selected to represent phylogenetic relationships among taxa in *Arthrographis*. Estimated base frequencies were as follows: A = 0.202, C = 0.318, G = 0.279, T = 0.201; substitution rates AC = 0.89564, AG = 2.18292, AT = 0.86983, CG = 1.56092, CT = 6.16286, GT = 1.00000. For BI analysis, the best-fit model identified for the dataset was GTR+F+I+G4. The phylogenetic tree was consistent and strongly supported in branches based on ML and BI analyses. Phylogenetic analyses demonstrated that the genus *Arthrographis* formed a distinct clade (50% MLBS and 0.95 BYPP) and appeared to be separated from *A. grakistii* Giraldo López & Hern.-Restr. (CBS 145529, JW199018, JW22015, JW22019, CBS 145530, JW209003, JW209002, JW49012, JW49011, and JW180011) and *A. longispora* A. Giraldo, Deanna A. Sutton, Cano & Guarro (CBS 135935 and CBS 145528). The four newly generated strains (GZUIFR 25.211, GZUIFR 25.212, GZUIFR 25.213, and GZUIFR 25.214) shared the same branch length with a high support value (99% MLBS and 1.00 BYPP; Fig. 3). Therefore, *Arthrographis guizhouensis* sp. nov. is introduced herein to the genus *Arthrographis*.

### Taxonomy

#### *Kickxellomycotina* Benny

***Kickxellomycetes* Tedersoo, Sánch.-Ram., Kõljalg, M.M. Bahram, M. Döring, Schigel, T.W. May, M. Ryberg & Abarenkov**

***Spiromycetales* Doweld**

##### Spiromycetaceae

Taxon classificationFungiKickxellalesSpiromycetaceae

Doweld

BF8DFEBC-AFBB-5E6A-919D-EB7C154122B0

###### Notes.

Doweld (2014) established *Spiromycetales* Doweld to accommodate a new family, *Spiromycetaceae* Doweld, and designated *Spiromyces* R.K. Benj. as the type genus. Species of *Spiromycetaceae* are characterized by sessile or stalked sporocladia, and sporocladia produce several pedicellate merosporangia by budding (Doweld 2014). Recently, only two genera have been listed in this family, comprising *Mycoëmilia* Kurihara, Degawa & Tokum. and *Spiromyces* R.K. Benj. (the type genus) (Ri and Degawa 2025). *Mycoëmilia* was introduced by Kurihara et al. (2004) and was initially accommodated in *Kickxellales* based on morphology, with the type species *Mycoëmilia scoparia* Kurihara, Degawa & Tokum. Later, based on LSU and SSU sequence analysis, Chuang et al. (2017) revealed that members of *Mycoëmilia* and *Spiromyces* formed a sister clade in the phylogenetic tree. Subsequently, Reynolds et al. (2023) found that *Mycoëmilia* and *Spiromyces* were placed in a clade of *Spiromycetales*, excluding *Kickxellales*, by multi-gene phylogenetic analysis. Hence, these two genera are accepted in *Spiromycetaceae*, *Spiromycetales*. Species of *Spiromycetaceae (Spiromycetales)* are commonly known as *M. scoparia*, *S. minutus*, and *S. aspiralis*.

##### Spiromyces

Taxon classificationFungiKickxellalesKickxellaceae

R.K. Benj.

BA9BCABE-1AA8-5848-92B5-C8025FF2E8D1

###### Notes.

Based on morphological characteristics, *Spiromyces* was proposed by Benjamin (1963) with *Spiromyces minutus* R.K. Benj. as the type species and was initially accommodated in *Kickxellaceae (Kickxellales)*. Subsequently, O’Donnell et al. (1998) demonstrated that *S. aspiralis* Benny & R.K. Benj. and *S. minutus* formed a distinct clade, which was separated from other members of *Kickxellales* in the phylogenetic tree of the 18S rDNA sequence analysis. Species of *Spiromyces* are characterized by erect or ascending sporophores, sessile or stalked sporocladia producing several pedicellate merosporangia by budding, and globose to slightly ovoid merosporangia. Members of *Spiromyces* were collected multiple times on mouse dung in Japan, Pakistan, and southern California (O’Donnell et al. 1998).

##### Spiromyces sinensis

Taxon classificationFungiKickxellalesKickxellaceae

H.Y. Wang & Y.F. Han sp. nov.

B2269A53-14B6-561D-90FB-41B453D0382D

862137

Fig. 4

###### Etymology.

Referring to China where the species was isolated.

###### Type.

China • Guizhou Province, Tongren City, tea garden (28°2'7"N, 108°59'58"E), rhizosphere soil of tea plant, April 2025, Haiyan Wang, ex-type culture GZUIFR 25.271, dried holotype GZAC 25.271.

###### Description.

Rhizosphere fungi associated with the tea plant. The fungus sporulated on PDA within 14 days of incubation at 25 °C. ***Hyphae*** hyaline, septate, 1.0–3.5 μm diam. ***Sporophores*** absent or erect, arising directly tips of hyphae. ***Sporocladia*** reduced to enlarged cells from the apex of hyphae, producing a membraneous remnant of the pedicel after spores falling off at maturity. ***Spores*** globose, abundant, rough-walled, hyaline, with pyramidal on the surface, 3.0–4.5 μm (av. 3.82 μm, *n* = 30), arising mostly directly from hyphae or a few from tips of hyphae enlarged cells, falling off at maturity. ***Zygospores*** unknown. ***Chlamydospores*** ovoid, abundant, thick-walled, 6.5–19.0 × 4.0–15.0 μm (av. 11.49 × 6.93 μm, *n* = 10).

**Figure 4.**
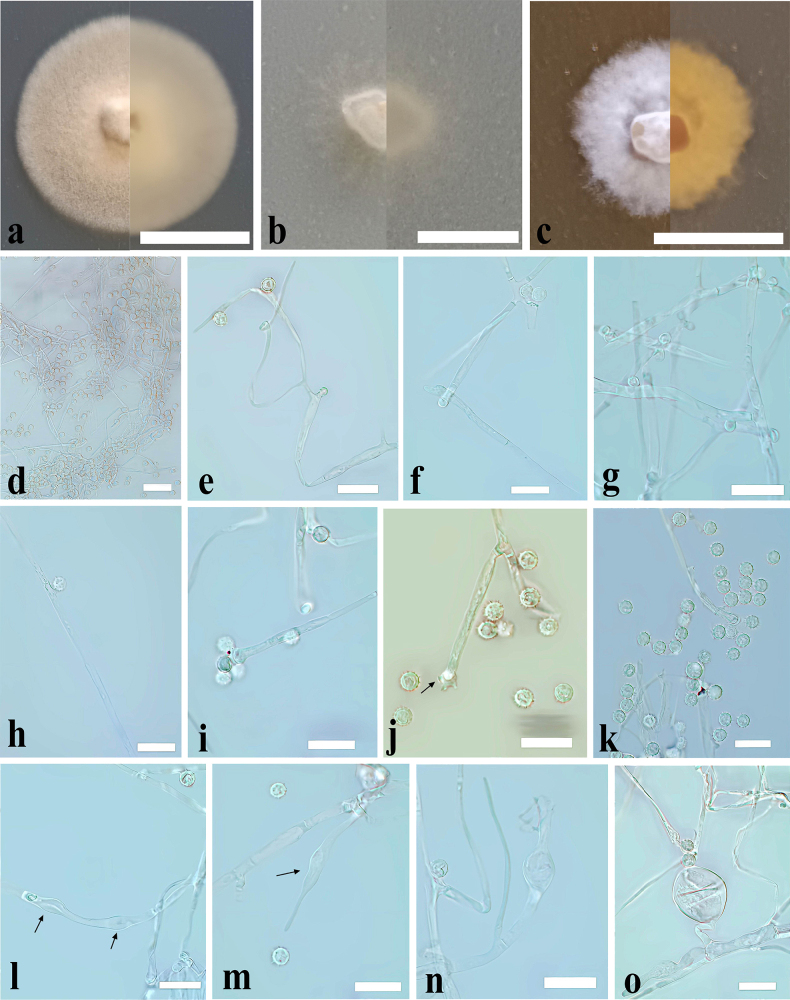
Morphological characteristics of *Spiromyces sinensis* (GZAC 25.271, holotype). **a–c**. Front and reverse of colony on PDA (**a**), OA (**b**), and MEA (**c**) after 14 d at 25 °C; **d**. Dense spores from hyphae; **e–g**. Young spores; **h, i**. Spores arising mostly directly from hyphae and from tips of hyphae; **j**. A remnant of the pedicel left behind after spore dehiscence; **k**. Mature spores; **l, m**. Young chlamydospores; **n, o**. Mature chlamydospores. Scale bars: 10 mm (**a–c**); 20 μm (**d**); 10 μm (**e–o**).

###### Culture characteristics.

Colony on PDA, after 14 days of incubation at 25 °C, reaching up to 20–25 mm diam., dense, villiform, margins entire, mycelia creamy white to gray, exudates and diffusible pigments absent, reverse, creamy white to gray, margin entire. Colony on MEA, reaching up to 10–15 mm diam., thick, fluffy, flocculence, margins entire, mycelia white, exudates and diffusible pigments absent, reverse, margins entire, white. Colony on OA, reaching up to 10–15 mm diam., thin, fluffy, flocculence, margin partial, mycelia gray, exudates and diffusible pigments absent, reverse, gray, margin partial.

###### Additional specimens examined.

China • Guizhou Province, Tongren City, Tea Garden (28°2'7"N, 108°59'58"E), rhizosphere soil of tea plant, April 2025, living cultures GZUIFR25.272; *ibid*., GZUIFR25.273.

###### Notes.

BLASTn analysis with the ITS, LSU, and SSU sequences of *Spiromyces sinensis* showed similarities to *S. minutus* of 95.92% with 28 gaps, 89.26% with 3 gaps, and 97.49% with 6 gaps, respectively. Phylogenetically, the three strains (GZUIFR 25.271, GZUIFR 25.272, and GZUIFR 25.273) can apparently be distinguished from *S. minutus*, *S. aspiralis*, and *Mycoëmilia scoparia* in *Spiromycetaceae (Spiromycetales)* and clustered in a single clade with a high support value (100% MLBS and 1.00 BYPP; Fig. 1). In the morphological characteristics, the three strains of *S. sinensis* have globose spores with pyramidal projections on the surface, arising directly from hyphae or enlarged cells, and ovoid chlamydospores, without zygospores. *Spiromyces sinensis* differed from *S. minutus* by producing ovoid and spinose mitospores, with globose zygospores, and without chlamydospores (Benjamin 1963; O’Donnell et al. 1998). *Spiromyces sinensis* differed from *S. aspiralis* in its presence of subglobose and warted mitospores, and absence of chlamydospores and zygospores (O’Donnell et al. 1998). *Spiromyces sinensis* can be distinguished from *Mycoëmilia scoparia* by its fusiform spores, spherical zygospores, and absence of chlamydospores (Kurihara et al. 2004). Therefore, *S. sinensis* is introduced as a new species.

#### *Sordariomycetes* O.E. Erikss. & Winka

***Coniochaetales* Huhndorf, A.N. Mill. & F.A. Fernández**

##### Coniochaetaceae

Taxon classificationFungiConiochaetalesConiochaetaceae

Malloch & Cain

A7EAB67C-8095-50D0-AC63-F976776C00F4

###### Notes.

*Coniochaetaceae* Malloch & Cain was proposed by Malloch and Cain (1971) to contain *Coniochaetidium* Malloch & Cain and *Coniochaeta* (Sacc.) Cooke, with *Coniochaeta* as the type genus. The members of *Coniochaetidium* were mostly re-evaluated and transferred to the genus *Coniochaeta*. Meanwhile, *Coniochaetidium* is regarded as a synonym of *Coniochaeta* (García et al. 2006). Currently, two genera are accommodated in this family, *Barrina* A.W. Ramaley and *Coniochaeta* (Silva et al. 2023). The genus *Barrina* was established with *B. polyspora* A.W. Ramaley as the type species (Ramaley 1997). Currently, the genus *Barrina* accommodates only this single species. The genus *Coniochaeta* differed from the sexual morph of *Barrina* in having immersed ascomata, cylindrical to fusoid asci, and ellipsoid-fusoid ascospores (Ramaley 1997; Silva et al. 2023).

##### Coniochaeta

Taxon classificationFungiConiochaetalesConiochaetaceae

(Sacc.) Cooke

201A9F2E-734D-5CBF-9E4E-C506745B20E5

###### Notes.

The genus *Coniochaeta* was introduced based on *C. ligniaria* (Grev.) Massee as the type species (García et al. 2006; Silva et al. 2023). The sexual morph of *Coniochaeta* is characterized by superficial or semi-immersed ascomata, cylindrical, clavate, subglobose, or globose asci, and narrowly ellipsoid to fusoid, broadly ellipsoidal to globose ascospores (García et al. 2006; Silva et al. 2023). The asexual morph of *Coniochaeta* is characterized by phialides with very short lateral necks, periclinal wall thickening, and flaring collarettes (Gams 2000; Weber 2002; Silva et al. 2023). Meanwhile, the genus *Coniochaeta* has various shapes of conidia, such as ellipsoidal to cylindrical, ellipsoid, ellipsoidal, cylindrical or curved, and bacilliform to allantoid (Arnold et al. 2021; Kabtani et al. 2022; Silva et al. 2023; Crous et al. 2025). Presently, 134 species of *Coniochaeta* are recorded in Index Fungorum (2025) (published on the Internet at http://www.indexfungorum.org, The Royal Botanic Gardens, Kew. [Retrieved 16 March 2026]).

##### Coniochaeta guizhouensis

Taxon classificationFungiConiochaetalesConiochaetaceae

H.Y. Wang & Y.F. Han sp. nov.

5491915B-B25E-5195-A39A-8B48B0EC53EE

862138

Fig. 5

###### Etymology.

The epithet “guizhouensis” refers to the locality, Guizhou Province, where the type was isolated.

###### Type.

China • Guizhou Province, Tongren City, Tea Garden (27°35'39"N, 108°11'41"E), rhizosphere soil of tea plant, April 2025, Haiyan Wang, ex-type culture GZUIFR25.151, dried holotype GZAC 25.151.

###### Description.

Rhizosphere fungi associated with the tea plant. The fungus sporulated on PDA medium after 14 days of incubation at 25 °C. ***Hyphae*** septate, hyaline, smooth, thick-walled, simple to branched, anastomosis observed, 1.0–4.0 μm wide. ***Conidiophores*** reduced to conidiogenous cells. ***Conidiogenous cells*** lateral or terminal, holoblastic, monophialidic, ampulliform phialides, with occasional cylindrical, solitary, septate, unbranched, straight, mostly flexuous at the apex, rarely the dehiscent apical, 1.0–20.5 × 1.0–3.0 µm (av. 8.61 × 1.91 µm, *n* = 30). ***Conidia*** abundant, hyaline, smooth-walled, aseptate, mostly ellipsoidal to oblong with round ends or one end slightly acute, arranged in slimy heads, 1.0–9.0 × 1.0–2.5 μm (av. 4.15 × 1.64 µm, *n* = 30), rare yeast-like cells observed (producing secondary conidia resembling budding yeasts). ***Chlamydospores*** abundant, globose or subglobose, smooth, thick-walled, 2.5–10.0 μm.

**Figure 5.**
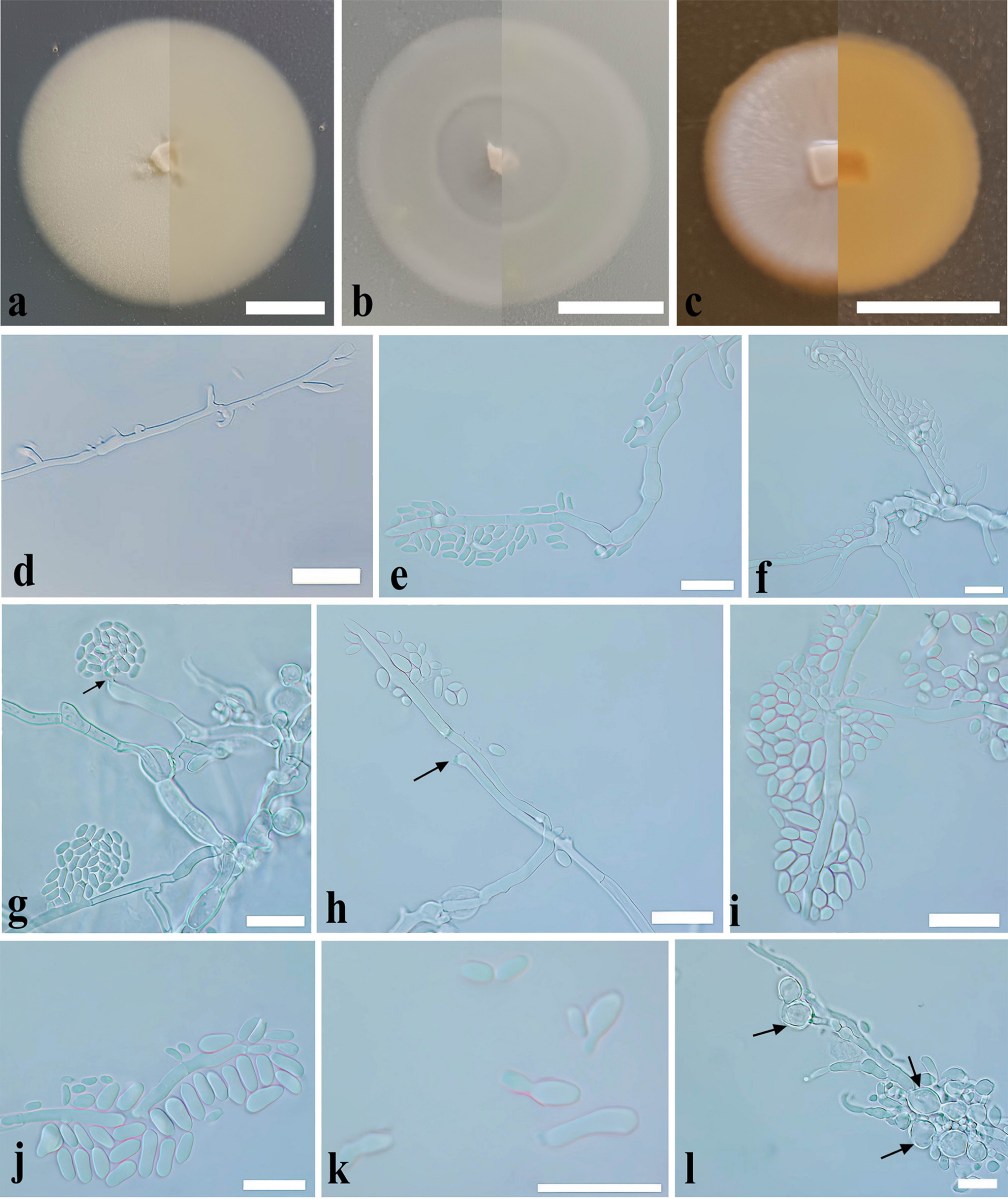
Morphological characteristics of *Coniochaeta guizhouensis* (GZAC 25.151, holotype). **a–c**. Front and reverse of colony on PDA (**a**), OA (**b**), and MEA (**c**) after 14 d at 25 °C; **d**. Phialides and conidiogenous cells arising directly from hyphae or the apex of hyphae; **e, f**. Phialides, conidiogenous cells, and conidia; **g, h**. Dehiscent apex of conidiogenous cells; **i, j**. Conidia; **k**. Yeast-like cells; **l**. Chlamydospores. Scale bars: 10 mm (**a–c**); 20 μm (**d**); 10 μm (**e–l**).

###### Sexual morph.

Not observed.

###### Culture characteristics.

Colony on PDA, after 14 days of incubation at 25 °C, reaching up to 35–40 mm diam., short villiform with dust, flat, creamy white, exudates and diffusible pigments absent, reverse creamy white, regular in the margin; Colony on MEA, reaching up to 15–20 mm diam., thick, moist, villiform, creamy white, exudates and diffusible pigments absent, reverse pale yellow, regular in the margin; Colony on OA, reaching up to 30–35 mm diam., butyrous, with rings, gray white, exudates and diffusible pigments absent, reverse gray white, regular in the margin.

###### Additional specimens examined.

China • Guizhou Province, Tongren City, Tea Garden (27°35'39"N, 108°11'41"E), rhizosphere soil of tea plant, April 2025, living cultures GZUIFR25.152; *ibid*., GZUIFR 25.153.

###### Notes.

BLASTn analysis with the ITS and LSU sequences of *Coniochaeta guizhouensis* showed similarities to *C. luteorubra* of 94.86% with 4 gaps and 99.21% without gaps, respectively. Phylogenetically, the three strains of the new species (GZUIFR 25.151, GZUIFR 25.152, and GZUIFR 25.153) clustered in a single subclade with a high support value (100% MLBS and 1.00 BYPP; Fig. 2). In the phylogenetic tree, *C. guizhouensis* has a close relationship with *Coniochaeta fodinicola* Vázq.-Camp., *C. queenslandica* Y.P. Tan & Bishop-Hurley, and *C. australiensis* Y.P. Tan & Bishop-Hurley. However, the new species differed from these three species in having conidiogenous cells mostly flexuous at the apex, abundant chlamydospores (2.5–10.0 μm diam.), and rare yeast-like cells. The new species can be distinguished from *C. fodinicola*, which has rare chlamydospores (7–8 μm diam.), abundant yeast-like cells, and no flexuous conidiogenous cells at the apex (Vázquez-Campos et al. 2014). Moreover, *C. fodinicola* has globose to subglobose perithecia and a mycelial swirl, but these two characteristics were not observed in the three strains of the new species. To date, the morphological descriptions of two species, *C. queenslandica* and *C. australiensis*, are unavailable, so it is impossible to compare them with the new species in terms of morphological characteristics in this paper (Tan 2023). Therefore, *Coniochaeta guizhouensis* is introduced as a new species.

#### *Dothideomycetes* O.E. Erikss. & Winka

***Eremomycetales* Crous, Spatafora, Haridas & I.V. Grig**.

##### Eremomycetaceae

Taxon classificationFungiEremomycetalesEremomycetaceae

Malloch & Cain

F5067FD2-19DB-5CAE-999A-5ED197A8B917

###### Notes.

*Eremomycetaceae*, only having the sexual morph, was proposed by Malloch and Cain (1971), with *Eremomyces* Malloch & Cain as the type genus. The sexual morph of the family was characterized by subglobose to globose ascocarps with a single cavity, subglobose to clavate asci, and one-celled ascospores (Malloch and Cain 1971). Meanwhile, the family has *arthrographis*-like or *trichosporiella*-like asexual morphs (Giraldo et al. 2014). Members of this family are widely distributed, such as in America, Canada, China, India, Kenya, and Tanzania, and have been found in air, compost, dung, marine sediments, soil, and wood, and from opportunistic infections in humans (Malloch and Cain 1971; Giraldo et al. 2014; Li et al. 2022). Giraldo et al. (2014) demonstrated that the family *Eremomycetaceae* included the genera *Arthrographis*, *Rhexothecium* Samson & Mouch., and *Eremomyces* based on the combined ITS, ACT, and CHS1 sequence dataset. The genus *Rhexothecium* has sexual and asexual morphs, but the genus *Eremomyces* only has the sexual morph (Malloch and Cain 1971; Samson and Mouchacca 1975). A few species of the genus *Arthrographis* have both sexual and asexual morphs, such as *Arthrographis curvata* A. Giraldo, Gené, Deanna A. Sutton & Cano, and most species only have an asexual morph (Giraldo et al. 2014). Currently, the family includes three genera: *Arthrographis*, *Eremomyces*, and *Rhexothecium* (Hyde et al. 2024).

##### Arthrographis

Taxon classificationFungiEremomycetalesEremomycetaceae

G. Cochet

BC8B01A6-4F69-5ED1-809C-95EF708E469C

###### Notes.

The genus *Arthrographis* was established by Cochet (1939), with *A. langeronii* G. Cochet as the type species. Due to a lack of a Latin diagnosis, it did not conform to the International Code of Botanical Nomenclature (ICBN), so it was regarded as invalid (Giraldo et al. 2014; Li et al. 2022). Later, based on morphological characteristics, Sigler and Carmichael (1976) introduced *Arthrographis kalrae* (basionym: *Oididendron kalrae* R.P. Tewari & Macph.) and *A. cuboidea* (basionym: *Scytalidium cuboideum* (Sacc. & Ellis) Sigler & Kang) and validated the genus name (Sigler and Carmichael 1976). The asexual morph of *Arthrographis* species was characterized by a slow growth rate and by the presence of cylindrical arthroconidia released schizolytically from dendritic conidiophores (Sigler and Carmichael 1976; Giraldo et al. 2014). Based on multi-gene phylogeny and morphological characteristics, four new species, *A. chlamydospora* A. Giraldo, Deanna A. Sutton, Cano & Madrid, *A. curvata* A. Giraldo, Gené, Deanna A. Sutton & Cano, *A. globosa* A. Giraldo, Deanna A. Sutton, Cano & Guarro, and *A. longispora* A. Giraldo, Deanna A. Sutton, Cano & Guarro, were proposed by Giraldo et al. (2014). Recently, *A. grakistii* Giraldo López & Hern.-Restr. was reported as a new species by Hernández-Restrepo et al. (2020). *Arthrographis multiformispora* Xin Li, Y.F. Han & Z.Q. Liang was introduced by Li et al. (2022), while some species were transferred to other genera (Kang et al. 2010; Giraldo et al. 2014). Currently, the genus *Arthrographis* includes 11 species in Index Fungorum (2025) (published on the Internet at http://www.indexfungorum.org, The Royal Botanic Gardens, Kew. [Retrieved 16 March 2026]).

##### Arthrographis guizhouensis

Taxon classificationFungiEremomycetalesEremomycetaceae

H.Y. Wang & Y.F. Han sp. nov.

B0201324-70BB-51CD-8293-D747E23FAF34

862139

Fig. 6

###### Etymology.

The epithet “guizhouensis” refers to the locality, Guizhou Province, where the type was isolated and collected.

###### Type.

China • Guizhou Province, Tongren City, Tea Garden (28°02'47"N, 108°33'41"E), rhizosphere soil of tea plant, April 2025, Haiyan Wang, ex-type culture GZUIFR25.211, dried holotype GZAC 25.211.

###### Description.

Rhizosphere fungi associated with the tea plant. The fungus sporulated on PDA medium after 14 days of incubation at 25 °C. ***Vegetative hyphae*** septate, hyaline, thick-walled, simple to branched, 1.0–4.0 μm wide. ***Conidiophores*** reduced to conidiogenous hyphae. ***Conidiogenous hyphae*** hyaline, smooth-walled, mostly erect, clavate, aseptate, simple or unbranched, up to 40.0 μm long, forming arthroconidia from the top to the base. ***Arthrospores*** in single or branched chains, or solitary, unicellular, arising from conidiogenous hyphae or vegetative hyphae, abundant, hyaline, smooth-walled, ovoid and elliptical, lateral, terminal, 2.5–9.0 × 2.5–5.0 μm (av. 4.71 × 3.30 μm, *n* = 30). ***Chlamydospores*** globose or subglobose, smooth, abundant, unicellular, thick-walled, 5.0–9.0 μm.

**Figure 6.**
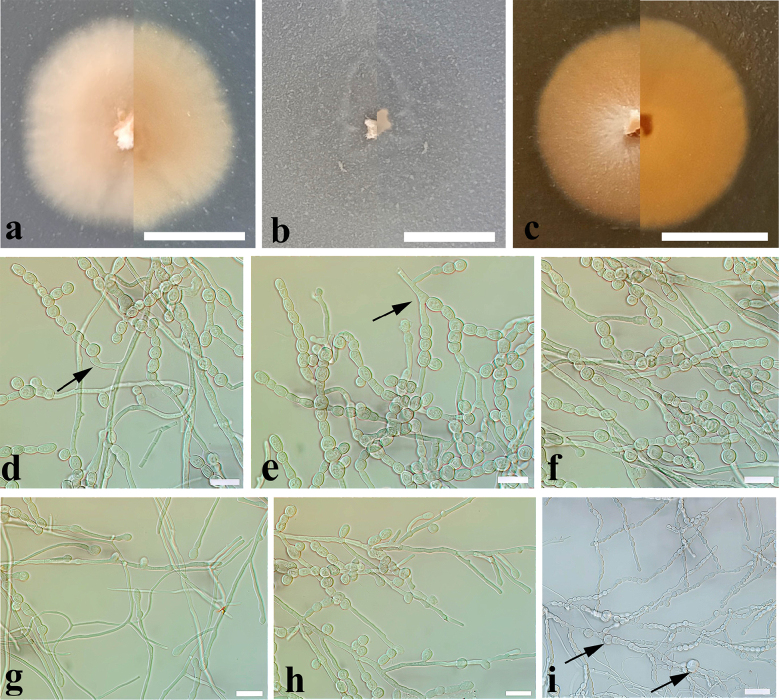
Morphological characteristics of *Arthrographis guizhouensis* (GZAC 25.211, holotype). **a–c**. Front and reverse of colony on PDA (**a**), OA (**b**), and MEA (**c**) after 14 d at 25 °C; **d–f**. Branched and unbranched conidiophores; **g, h**. Arthroconidia arising directly from the tip or middle of the hyphae; **i**. Chlamydospores. Scale bars: 10 mm (**a–c**); 10 μm (**d–h**); 20 μm (**i**).

###### Sexual morph.

Not observed.

###### Culture characteristics.

Colony on PDA, after 14 days of incubation at 25 °C, reaching up 20–25 mm diam., thick, short villiform, radial mycelium, fleshcolor at the center, creamy white at the edge, exudates and diffusible pigments absent, reverse creamy white, regular in the margin; Colony on MEA, reaching up 20–25 mm diam., villiform, moist, creamy white at the center, pale brown at the edge, exudates and diffusible pigments absent, reverse pale brown, regular in the margin; Colony on OA, reaching up 20–25 mm diam., thin, fluffy, flocculence, gray white, damp marks, reverse gray white, irregular in the margin.

###### Additional specimens examined.

China • Guizhou Province, Tongren City, Tea Garden (28°02'47"N, 108°33'41"E), rhizosphere soil of tea plant, April 2025, living cultures GZUIFR25.212; *ibids*., GZUIFR25.213, GZUIFR25.214.

###### Notes.

BLASTn analysis with the ITS sequences of *Arthrographis guizhouensis* showed similarity to *A. kalrae* with a percent similarity of 96.23% without gaps. The analysis with LSU sequences of *A. guizhouensis* showed similarity to *A. curvata* with a percent similarity of 99.03% without gaps, and the analysis with ACT sequences of *A. guizhouensis* showed similarity to *A. kalrae* with a percent similarity of 96.10% with 4 gaps. Phylogenetically, the four strains (GZUIFR 25.211, GZUIFR 25.212, GZUIFR 25.213, and GZUIFR 25.214) of the new species formed a monophyletic subclade with a high support value (1.00 BYPP and 99% MLBS; Fig. 3) and had a close relationship with *A. grakistii* Giraldo López & Hern.-Restr. and *A. longispora* A. Giraldo, Deanna A. Sutton, Cano & Guarro. However, they were obviously different in morphological characteristics. *Arthrographis guizhouensis* does not produce conidiophores or has conidiophores reduced to conidiogenous hyphae, has solitary or branched chains, ovoid and elliptical arthrospores from conidiogenous hyphae or vegetative hyphae, and has no synasexual morph, which can be distinguished from *A. longispora* and *A. grakistii*. *Arthrographis longispora* has cylindrical arthroconidia with truncate or rounded ends (Giraldo et al. 2014). *Arthrographis grakistii* has semi-macronematous or micronematous conidiophores and cylindrical or cuboid arthroconidia (Hernández-Restrepo et al. 2020). Therefore, *Arthrographis guizhouensis* is introduced as a new species.

## Discussion

To date, the number of fungal species described by mycologists remains very limited, and many fungal taxonomists continue to make considerable efforts. In recent years, they have tended to use various methods to explore fungal species in specific habitats. For instance, Yang et al. (2023) conducted a comprehensive study on freshwater fungi found in karst landscapes in China and Thailand. Using different modified media, Zhang et al. (2024) attempted to maximize the number of fungi isolated from soil samples collected from the green belts of Guizhou Wildlife Park in southwest China. Bao et al. (2025) isolated fungi using single-spore isolation to explore the biodiversity of lignicolous freshwater fungi from the Nanpan River Basin in Guizhou and Guangxi Provinces, China. Chen et al. (2025) reported the cryptic diversity of cordyceps-like fungi in karst regions of Guizhou Province, China. Despite these efforts, studies on culturable fungi from the rhizosphere soil of tea plants remain limited. According to a comprehensive analysis and market trend report of the global tea industry, China is the country with the largest tea tree planting area in the world. In this study, rhizosphere soil fungi of tea plants were isolated using traditional culturable methods in Guizhou Province, a representative area in China. Three modified culture media—Martin’s medium, Sabouraud’s dextrose agar, and Sabouraud’s dextrose agar yeast extract (Zhang et al. 2024)—were employed to maximize the isolation of fungi from the rhizosphere soil of tea plants. During the fungal diversity survey, three new species, *Arthrographis guizhouensis*, *Coniochaeta guizhouensis*, and *Spiromyces sinensis*, were isolated and introduced herein. This study expands the members of these three genera, *Arthrographis*, *Coniochaeta*, and *Spiromyces*, and provides new species resources for future research related to tea plants.

Phylogenetic analysis 1 revealed that strains of the new species are close to *Spiromyces minutus* in *Spiromycetales* and form a distinct clade in the phylogenetic tree (Fig. 1). Notably, in Index Fungorum, two genera, *Spiromyces* and *Mycoëmilia*, are included in *Kickxellaceae*, *Kickxellales*. However, through phylogenetic analysis, many reports revealed that these two genera are clearly separated from the members of *Kickxellaceae*, *Kickxellales*, and are included in *Spiromycetaceae*, *Spiromycetales* (Ri et al. 2022; Reynolds et al. 2023; Ri and Degawa 2025). The present phylogenetic analysis is consistent with those studies. Currently, three species, *S. aspiralis*, *S. minutus*, and *S. sinensis*, are included in the genus *Spiromyces*. The report of the new species *S. sinensis* enriches the species diversity of *Spiromycetaceae*, *Spiromycetales*, and provides the basic gene sequence for its future study. Members of *Spiromycetales* are generally regarded as having a saprophytic lifestyle (Ri and Degawa 2025). Previously, *Spiromyces* spp. have been isolated from mouse dung (O’Donnell et al. 1998), and *Mycoëmilia scoparia* from moist chamber soil (Kurihara et al. 2004). In this study, three strains of the new species *S. sinensis* were isolated from tea rhizosphere soil, and the biological functions of these strains require further investigation.

Phylogenetic analysis 2 revealed the evolutionary relationship between the new species and members of the genus *Coniochaeta*. Three strains of the new species *C. guizhouensis* are close to *C. australiensis* and *C. queenslandica* in the phylogenetic tree (Fig. 2). Unfortunately, these two species lack morphological characteristics. The LSU sequence of *C. queenslandica* (BRIP 74376a) is also unavailable. It is expected that morphological feature descriptions and the sequence can be supplemented in future taxonomic work. Some species of the genus *Coniochaeta* have sexual and asexual morphs (Gams 2000; Weber 2002; García et al. 2006; Silva et al. 2023). However, the sexual morph of the new species was not discovered. In further research, it may be necessary to attempt special methods to cultivate this species and explore its sexual morph. Most species of *Coniochaeta* display similar flat and moist colonies (Kabtani et al. 2022), and only *C. mutabilis* presents low aerial growth (Drees et al. 2007). The new species *C. guizhouensis* also displays the same flat and moist colonies. The genus *Coniochaeta* exhibits diverse ecological niches, including associations with plants, animal dung, water, wood, food, human pathogens, mine raffinate, and horticultural substrates (Damm et al. 2010; Khan et al. 2013; Vázquez-Campos et al. 2014; Kabtani et al. 2022; Crous et al. 2025). The genus also includes endophytic species, such as *C. ligniaria*, *C. endophytica*, *C. salicifolia*, and *C. monsterae* (Silva et al. 2023). However, *C. monsterae* does not cause any disease symptoms in its host (Silva et al. 2023). Some species of the genus *Coniochaeta*, *C. mutabilis* and *C. hoffmannii*, are the most familiar human pathogens, which are the most widespread and commonly encountered in human samples and severe infections (Kabtani et al. 2022). In this study, three strains of the new species *C. guizhouensis* were isolated from tea rhizosphere soil, but it is currently unclear whether they cause any disease symptoms in tea plants.

Phylogenetic analysis 3 revealed the evolutionary relationship between the new species and members of the genus *Arthrographis* (Fig. 3). In the phylogenetic tree, the genus *Arthrographis* comprises nine accepted species, including the novel species *A. guizhouensis*. Members of the genus *Arthrographis* have been isolated from different environments, which reflects their ecological versatility and adaptive potential. *Arthrographis* species have been isolated from air, compost, marine sediments, soil, wood, clinical specimens, and opportunistic infections in humans (Giraldo et al. 2013; Giraldo et al. 2014; Li et al. 2022). *Arthrographis* spp. are often considered the source of reported opportunistic infections. For example, the type culture of *A. kalrae* was isolated from the sputum of a man with an ill-defined respiratory infection in India (Tewari and Macpherson 1971). *Arthrographis kalrae* was reported to cause pansinusitis and meningitis (Chin-Hong et al. 2001). Meanwhile, Yoshitsugu and Masaki (2010) reported a rare case of onychomycosis due to *A. kalrae*. *Arthrographis multiformispora* was obtained from soil beside a park road by a method specifically designed for isolating keratinophilic fungi, but it is uncertain whether *A. multiformispora* is an opportunistic pathogen that infects the skin (Li et al. 2022). In this study, four strains of *A. guizhouensis* were isolated from tea rhizosphere soil. Whether the new species is a source of opportunistic infections remains to be further studied.

In conclusion, this study described and proposed three new species from tea rhizosphere soil based on morphological characteristics and multi-gene phylogeny. Meanwhile, more novel taxa are expected to be discovered and reported from tea rhizosphere soil, which will provide opportunities for the future exploration of beneficial fungal species associated with the tea plant.

## Supplementary Material

XML Treatment for Spiromycetaceae

XML Treatment for Spiromyces

XML Treatment for Spiromyces sinensis

XML Treatment for Coniochaetaceae

XML Treatment for Coniochaeta

XML Treatment for Coniochaeta guizhouensis

XML Treatment for Eremomycetaceae

XML Treatment for Arthrographis

XML Treatment for Arthrographis guizhouensis

## Additional information

### Artificial Intelligence (AI) use

The authors accept full responsibility for the content of the manuscript, including the disclosure of any use of AI.

No AI tools were used in the preparation of this manuscript.

### Author ORCIDs

H.-Y. Wang https://orcid.org/0000-0001-9190-0490

H.-Q. Qu https://orcid.org/0009-0000-5590-4072

Y.-F. Zhao https://orcid.org/0009-0003-8950-6891

K.-Y. Song https://orcid.org/0009-0002-7274-6277

H.-L. Zhang https://orcid.org/0009-0008-2413-3023

Y.-L. Wang https://orcid.org/0009-0000-3118-4867

C.-B. Dong https://orcid.org/0000-0001-7074-5700

Y.-W. Zhang https://orcid.org/0000-0003-1251-5821

W.-H. Chen https://orcid.org/0000-0001-7240-6841

Y.-F. Han https://orcid.org/0000-0002-8646-3975

### Data availability

All of the data that support the findings of this study are available in the main text or Supplementary Information.

## Supplementary materials

Supplementary material 1 GenBank numbers Data type docx Explanation note **table S1**. Strains of *Kickxellomycotina*, including the new species and corresponding GenBank numbers included in the phylogenetic analyses. **table S2**. Strains of *Coniochaeta* and corresponding GenBank numbers included in the phylogenetic analyses. **table S3**. Strains of *Arthrographis* and corresponding GenBank numbers included in the phylogenetic analyses. https://binary.pensoft.net/file/1675596This dataset is made available under the Open Database License (http://opendatacommons.org/licenses/odbl/1.0/). The Open Database License (ODbL) is a license agreement intended to allow users to freely share, modify, and use this Dataset while maintaining this same freedom for others, provided that the original source and author(s) are credited. Hai-Yan Wang, Hong-Qin Qu, Yu-Feng Zhao, Ke-Yun Song, Hai-Long Zhang, Yan-Ling Wang, Chun-Bo Dong, Yan-Wei Zhang, Wan-Hao Chen, Yan-Feng Han
