# Supplementary material for: Three new species (Coniochaetales, Eremomycetales, Spiromycetales) isolated from rhizosphere soil of tea plant (Camellia sinensis) in Guizhou, China
Source: MycoKeys. 2026 Jun 10;134:1–25. doi: 10.3897/mycokeys.134.186271 (PMC13276500; doi:10.3897/mycokeys.134.186271)
Supplement: Supplementary material 1 — GenBank numbers [file mycokeys-134-001-s001.docx]

**Supplementary materials**
**Table S1.** Strains of *Kickxellomycotina*, including the new species and corresponding GenBank numbers included in the phylogenetic analyses.

| **Species** | **Strains** | **ITS** | **LSU** | **SSU** | **Reference** |
| --- | --- | --- | --- | --- | --- |
| *Barbatospora ambicaudata* | TN-49-W4a | KC297566 | KF848899 | KC297614 | Ri et al. 2022 |
| ***Spiromyces sinensis*** | **GZUIFR 25.271 T** | **PX711160** | **PX711163** | **PX705989** | **This study** |
| ***Spiromyces sinensis*** | **GZUIFR 25.272** | **PX711161** | **PX711164** | **PX705990** | **This study** |
| ***Spiromyces sinensis*** | **GZUIFR 25.273** | **PX711162** | **PX711165** | **PX705991** | **This study** |
| *Capniomyces stellatus* | MIS-21-127 | – | EF396194 | EF396191 | Ri et al. 2022 |
| *Caudomyces* sp. | UT-1-W16a | KF848859 | JX155646 | JX155620 | Ri et al. 2022 |
| *Coemansia braziliensis* | NRRL-1566 | KF848850 | AF031069 | AF007532 | Ri et al. 2022 |
| *Dipsacomyces acuminosporus* | NRRL-2925 | – | KF848901 | – | Ri et al. 2022 |
| *Harpellomyces montanus* | TN-22-W5B | – | JQ302961 | JQ302887 | Ri et al. 2022 |
| *Kickxella alabastrina* | NRRL-2693 | – | KF848900 | – | Ri et al. 2022 |
| *Legeriomyces minae* | PEI-X-6 | – | JX155648 | JX155622 | Ri et al. 2022 |
| *Linderina macrospora* | BCRC 31802 | JN942679 | GU138617 | JQ004924 | Ri et al. 2022 |
| *Linderina pennispora* | NRRL-3781 | – | KF848902 | – | Ri et al. 2022 |
| *Martensiomyces pterosporus* | NRRL-2642 | KF848853 | – | AF007539 | Ri et al. 2022 |
| *Mycoëmilia scoparia* | NBRC 100468 | – | NG_071233 | – | Ri et al. 2022 |
| *Myconymphaea yatsukahoi* | NBRC 100467 | – | NG_071232 | NG_070865 | Ri et al. 2022 |
| *Orphella dalhousiensis* | NS-34-W16 | KF848856 | – | – | Ri et al. 2022 |
| *Orphella haysii* | AFTOL-ID 1062 | AY997068 | DQ273830 | DQ322626 | Ri et al. 2022 |
| *Orphella pseudohiemalis* | OR-14-W25A | – | MG845408 | MG845403 | Ri et al. 2022 |
| *Pinnaticoemansia coronantispora* | NBRC 100470 | – | NG_071234 | NG_070867 | Ri et al. 2022 |
| *Piptocephalis corymbifera* | NRRL-2385 | NR_119548 | NG_027616 | NG_017192 | Ri et al. 2022 |
| *Ramicandelaber brevisporus* | NBRC 100469 | – | AB288001 | AB287987 | Ri et al. 2022 |
| *Ramicandelaber fabisporus* | BCRC 34358 | NR_154391 | NG_060077 | NG_070281 | Ri et al. 2022 |
| *Ramicandelaber longisporus* | ATCC 6175 | – | KF848913 | – | Ri et al. 2022 |
| *Rhopalomyces elegans* | AFTOL-ID 142 | – | DQ273795 | AY635834 | Ri et al. 2022 |
| *Smittium morbosum* | AUS-X-1 | KF848858 | KF848911 | AF277014 | Ri et al. 2022 |
| *Smittium mucronatum* | FRA-12-3 | JN943282 | KF848910 | AF277030 | Ri et al. 2022 |
| *Smittium simulii* | CAL-8-1 | JN943275 | JN940499 | JN940692 | Ri et al. 2022 |
| *Smittium simulii* | 41-1-6 | JN943276 | JN940498 | JN940693 | From NCBI |
| *Spirodactylon aureum* | NRRL 2810 | – | – | AF007541 | Ri et al. 2022) |
| *Spiromyces aspiralis* | NRRL-22631 | NR_119554 | NG_027560 | NG_017187 | Ri et al. 2022 |
| *Spiromyces aspiralis* | AFTOL-ID 185 | AY997090 | DQ273801 | – | From NCBI |
| *Spiromyces minutus* | NRRL-3067 | NR_119555 | NG_042415 | NG_061005 | Ri et al.(2022 |
| *Spiromyces minutus* | AFTOL-ID 327 | AY997091 | DQ273810 | – | From NCBI |
| *Unguispora grylli* | NBRC 116289 | PQ481193 | PQ481189 | PQ481185 | Ri et al. 2025 |
| *Unguispora rhaphidophoridarum* | NBRC 114906 | – | OK632445 | OK632441 | Ri et al. 2022 |
| *Zancudomyces culisetae* | COL-18-3 | – | DQ273773 | AF007540 | Ri et al. 2022 |

Note: T = ex-type; new isolates in this study are in bold; the hyphen “–” represents the absence of a GenBank record. ITS: internal transcribed spacer region and intervening 5.8S nrRNA; LSU: 28S large subunit; SSU: 18S small subunit nuclear rRNA gene.

**Table S2.** Strains of *Coniochaeta* and corresponding GenBank numbers included in the phylogenetic analyses.

| **Species** | **Strains** | **ITS** | **LSU** | **Reference** |
| --- | --- | --- | --- | --- |
| *Chaetosphaeria innumera* | SMH 2748 | AY906956 | AY017375 | Silva et al. 2023 |
| *Chaetosphaeria polygonalis* | KUNCC:23-13789 | PQ845785 | PV536238 | Silva et al. 2023 |
| *Coniochaeta acaciae* | MFLUCC 17-2298T | MG062735 | MG062737 | Silva et al. 2023 |
| *Coniochaeta africana* | CBS 120868T | GQ154539 | GQ154601 | Silva et al. 2023 |
| *Coniochaeta angustispora* | CBS 872.73 | MH860817 | MH872549 | Silva et al. 2023 |
| *Coniochaeta arenariae* | MFLUCC 18-0409 | MN047126 | MN017896 | Silva et al. 2023 |
| *Coniochaeta aurantiaca* | CGMCC 3.22339 T | NR_191236 | NG_243251 | Crous et al. 2025 |
| *Coniochaeta australiensis* | BRIP 74375a T | NR_191317 | NG_243408 | Crous et al. 2025 |
| *Coniochaeta baysunika* | MFLUCC 17-0830T | MG828880 | MG828996 | Silva et al. 2023 |
| *Coniochaeta* *baysunika* | TASM 6131 T | NR_157508 | – | Crous et al. 2025 |
| *Coniochaeta boothii* | CBS 381.74T | NR_159776 | AJ875226 | Silva et al. 2023 |
| *Coniochaeta canina* | R-4810 | JX481775 | JX481774 | Silva et al. 2023 |
| *Coniochaeta caraganae* | MFLUCC 18-0780 T | NR_185589 | NG_228849 | Crous et al. 2025 |
| *Coniochaeta cateniformis* | CBS 131709T | MH865902 | MH877340 | Silva et al. 2023 |
| *Coniochaeta cephalothecoides* | L821 | KY064029 | KY064030 | Silva et al. 2023 |
| *Coniochaeta cipronana* | CBS 144016T | NR_157478 | – | Silva et al. 2023 |
| *Coniochaeta coluteae* | MFLUCC 17-2299T | MG137251 | MG137252 | Silva et al. 2023 |
| *Coniochaeta corticalis* | JKI-GP-23-050 T | PV272673 | PV259250 | Crous et al. 2025 |
| *Coniochaeta cruciata* | FMR 7409 | – | AJ875222 | Silva et al. 2023 |
| *Coniochaeta cymbiformispora* | NBRC 32199 T | NR_175055 | LC146726 | Crous et al. 2025 |
| *Coniochaeta deborreae* | CBS 147215T | MW883413 | MW883808 | Silva et al. 2023 |
| *Coniochaeta decumbens* | CBS 153.42T | HE610337 | AF353597 | Silva et al. 2023 |
| *Coniochaeta dendrobiicola* | DLCCR7 | MK225602 | MK225603 | Silva et al. 2023 |
| *Coniochaeta discoidea* | CBS 158.80T | NR_159779 | AJ875230 | Silva et al. 2023 |
| *Coniochaeta discospora* | CBS 168.58 T | MH857740 | – | Crous et al. 2025 |
| *Coniochaeta elegans* | ARIZ FF0093 T | NR_177572 | – | Crous et al. 2025 |
| *Coniochaeta ellipsoidea* | CBS 137.68 T | MH859091 | MH870804 | Crous et al. 2025 |
| *Coniochaeta endophytica* | AEA 9094T | EF420005 | EF420069 | Silva et al. 2023 |
| *Coniochaeta euphorbiae* | 1001T | KP941076 | KP941075 | Silva et al. 2023 |
| *Coniochaeta extramundana* | CBS 247.77 T | MH861057 | MH872828 | Crous et al. 2025 |
| *Coniochaeta fasciculata* | CBS 205.38T | HE610336 | AF353598 | Silva et al. 2023 |
| *Coniochaeta fermentaria* | JKI-GP-22-032 T | PV272676 | PV259253 | Crous et al. 2025 |
| *Coniochaeta fibricola* | JKI-GP-22-031 T | PV272674 | – | Crous et al. 2025 |
| *Coniochaeta fibrosae* | CGMCC3.20304T | MW750760 | MW750758 | Silva et al. 2023 |
| *Coniochaeta* *fibrosae* | CX04D1 | MW750756 | MW750755 | Crous et al. 2025 |
| *Coniochaeta fodinicola* | CBS 136963T | JQ904603 | KF857172 | Silva et al. 2023 |
| *Coniochaeta gigantospora* | ILLS 60816T | JN684909 | – | Silva et al. 2023 |
| *Coniochaeta groatii* | JP5 T | OQ297064 | – | Crous et al. 2025 |
| ***Coniochaeta guizhouensis*** | **GZUIFR25.151 T** | **PX715493** | **PX715499** | **This study** |
| ***Coniochaeta guizhouensis*** | **GZUIFR25.152** | **PX715494** | **PX715500** | **This study** |
| ***Coniochaeta guizhouensis*** | **GZUIFR25.153** | **PX715495** | **PX715501** | **This study** |
| *Coniochaeta* *hansenii* | CBS 885.68 | – | AJ875223 | Silva et al. 2023 |
| *Coniochaeta hansenii* | RGM 3311 | OP962068 | OP962070 | Silva et al. 2023 |
| *Coniochaeta hoffmannii* | CBS 245.38T | HE610332 | AF353599 | Silva et al. 2023 |
| *Coniochaeta iranica* | CBS 139767T | KP941078 | KP941077 | Silva et al. 2023 |
| *Coniochaeta krabiensis* | MFLU 16-1230 | – | MN017892 | Silva et al. 2023 |
| *Coniochaeta leucoplaca* | CBS 486.73 | – | MH872465 | Silva et al. 2023 |
| *Coniochaeta ligniaria* | CBS 424.65T | MH858650 | AF353584 | Silva et al. 2023 |
| *Coniochaeta lignicola* | CBS 267.33T | HE610335 | FR691986 | Silva et al. 2023 |
| *Coniochaeta luteorubra* | CBS 131710T | HE610330 | HE610328 | Silva et al. 2023 |
| *Coniochaeta luteoviridis* | CBS 206.38T | HE610333 | AF353603 | Silva et al. 2023 |
| *Coniochaeta malacotricha* | F2107 | – | AF353590 | Silva et al. 2023 |
| *Coniochaeta marina* | MFLUCC 18-0408T | MK458764 | MK458765 | Silva et al. 2023 |
| *Coniochaeta massiliensis* | PMML0158 | OM366153 | – | Silva et al. 2023 |
| *Coniochaeta mongoliae* | CGMCC3.20250T | MW077645 | MW077646 | Silva et al. 2023 |
| *Coniochaeta monsterae* | RV01 | MZ648895 | MZ648891 | Silva et al. 2023 |
| *Coniochaeta monsterae* | RV02 | MZ648896 | MZ648892 | Silva et al. 2023 |
| *Coniochaeta montana* | ARIZ-SR0076 | MZ262414 | – | Crous et al. 2025 |
| *Coniochaeta mutabilis* | CBS 157.44T | NR_111519 | NG_042382 | Silva et al. 2023 |
| *Coniochaeta navarrae* | LTA3 | KU762326 | KU762326 | Silva et al. 2023 |
| *Coniochaeta* *navarrae* | CBS 141016 T | NR_154808 | - | Crous et al. 2025 |
| *Coniochaeta nepalica* | NBRC 30584T | LC146727 | LC146727 | Silva et al. 2023 |
| *Coniochaeta nivea* | ARIZ AK0926 T | NR_177571 | – | Crous et al. 2025 |
| *Coniochaeta notelaeae* | BRIP 66975a T | NR_200997 | – | Crous et al. 2025 |
| *Coniochaeta ornata* | FMR 7415T | – | AJ875228 | Silva et al. 2023 |
| *Coniochaeta ostrea* | CBS 507.70T | NR_159772 | AJ875227 | Silva et al. 2023 |
| *Coniochaeta palaoa* | ARIZ: AEANC0604 T | MZ241149 | - | Crous et al. 2025 |
| *Coniochaeta polymorpha* | CBS 132722T | NR121473 | HE863327 | Silva et al. 2023 |
| *Coniochaeta polysperma* | CBS 669.77 T | MH861109 | MH872868 | Crous et al. 2025 |
| *Coniochaeta prunicola* | CBS 120875T | GQ154540 | GQ154602 | Silva et al. 2023 |
| *Coniochaeta psammospora* | CBS 148.70 | MH859530 | MH871309 | Crous et al. 2025 |
| *Coniochaeta pulveracea* | CBS 114628 | MW883414 | GQ351560 | Crous et al. 2025 |
| *Coniochaeta punctulata* | FMR 7408 | – | AJ875231 | Silva et al. 2023 |
| *Coniochaeta queenslandica* | BRIP 74376a T | NR_191318 | – | Crous et al. 2025 |
| *Coniochaeta rankiniae* | BRIP 74950a T | NR_189971 | NG_242134 | Crous et al. 2025 |
| *Coniochaeta rhopalochaeta* | CBS 109872 T | NR_172554 | GQ351561 | Crous et al. 2025 |
| *Coniochaeta riskalishoyakubovii* | TASM 6166 T | NR_191221 | NG_243212 | Crous et al. 2025 |
| *Coniochaeta rosae* | MFLUCC 17-0810T | NR_157509 | NG_066204 | Silva et al. 2023 |
| *Coniochaeta salicifolia* | GS02.1.6 | MT573531 | MT525302 | Silva et al. 2023 |
| *Coniochaeta savoryi* | CBS 725.74T | MH860890 | AJ875229 | Silva et al. 2023 |
| *Coniochaeta simbalensis* | NFCCI 4236T | NR164024 | NG_068555 | Silva et al. 2023 |
| *Coniochaeta sinensis* | CGMCC3.20306T | MW422269 | MW422265 | Silva et al. 2023 |
| *Coniochaeta sordaria* | CBS 492.73 | – | MH878380 | Silva et al. 2023 |
| *Coniochaeta subcorticalis* | CBS 551.75 | – | AF353593 | Silva et al. 2023 |
| *Coniochaeta taeniospora* | CBS 141014T | KU762324 | KU762324 | Silva et al. 2023 |
| *Coniochaeta teitelbaumiae* | BRIP 72364b T | PQ882520 | PQ882520 | Crous et al. 2025 |
| *Coniochaeta tetraspora* | CBS 139.68 | MH859093 | MH870806 | Crous et al. 2025 |
| *Coniochaeta tritici* | IRAN 3464C T | MW432175 | – | Crous et al. 2025 |
| *Coniochaeta velutina* | CBS 121444 | GQ154544 | GQ154605 | Silva et al. 2023 |
| *Coniochaeta verticillata* | CBS 816.71T | NR_159774 | AJ875232 | Silva et al. 2023 |
| *Coniochaeta vineae* | KUMCC 17-0322T | NR_168225 | MN473512 | Silva et al. 2023 |
| *Coniochaeta weberae* | JKI-GP-23-051 T | PV272675 | PV259252 | Crous et al. 2025 |

Note: T = ex-type; new isolates in this study are in bold; the hyphen “–” represents the absence of a GenBank record. ITS: internal transcribed spacer region and intervening 5.8S nrRNA; LSU: 28S large subunit.

**Table S3.** Strains of *Arthrographis* and corresponding GenBank numbers included in the phylogenetic analyses.

| **Species** | **Strains** | **ITS** | **LSU** | **ACT** | **Reference** |
| --- | --- | --- | --- | --- | --- |
| *Arthrographis arxii* | CBS 203.78 T | GQ272638 | AB213426 | HG316563 | Li et al. 2022 |
| *Arthrographis chlamydospora* | CBS 135936 T | HG004554 | HG004543 | HG316560 | Li et al. 2022 |
| *Arthrographis curvata* | CBS 135934 T | HG004556 | HG004542 | HG316558 | Li et al. 2022 |
| *Arthrographis globosa* | UTHSC 11-757 T | HG004553 | HG004541 | HG316561 | Li et al. 2022 |
| *Arthrographis grakistii* | CBS 145529 | MN794359 | MN794336 | MN816497 | Li et al. 2022 |
| *Arthrographis grakistii* | JW22015 | MN794360 | MN794337 | MN816498 | Li et al. 2022 |
| *Arthrographis grakistii* | JW22019 | MN794361 | MN794338 | MN816499 | Li et al. 2022 |
| *Arthrographis grakistii* | JW49011 | MN794362 | MN794339 | MN816500 | Li et al. 2022 |
| *Arthrographis grakistii* | JW49012 | MN794363 | MN794340 | MN816501 | Li et al. 2022 |
| *Arthrographis grakistii* | JW180011 | MN794364 | MN794341 | MN816502 | Li et al. 2022 |
| *Arthrographis grakistii* | CBS 145530 | MN794365 | MN794342 | MN816503 | Li et al. 2022 |
| *Arthrographis grakistii* | JW190018 | MN794366 | MN794343 | MN816504 | Li et al. 2022 |
| *Arthrographis grakistii* | JW209002 | MN794367 | MN794344 | MN816505 | Li et al. 2022 |
| *Arthrographis grakistii* | JW209003 | MN794368 | MN794345 | MN816506 | Li et al. 2022 |
| ***Arthrographis guizhouensis*** | **GZUIFR25.211 T** | **PX775488** | **PX775494** | **PX811115** | **This study** |
| ***Arthrographis guizhouensis*** | **GZUIFR25.212** | **PX775489** | **PX775495** | **PX811116** | **This study** |
| ***Arthrographis guizhouensis*** | **GZUIFR25.213** | **PX775490** | **PX775496** | **PX811117** | **This study** |
| ***Arthrographis guizhouensis*** | **GZUIFR25.214** | **PX775491** | **PX775497** | **PX811118** | **This study** |
| *Arthrographis kalrae* | CBS 693.77 T | AB116536 | AB116544 | HG316544 | Li et al. 2022 |
| *Arthrographis kalrae* | JW 21004 | MN794369 | MN794346 | MN816507 | Li et al. 2022 |
| *Arthrographis kalrae* | CBS 145527 | MN794370 | MN794347 | MN816508 | Li et al. 2022 |
| *Arthrographis kalrae* | JW 21029 | MN794371 | MN794348 | MN816509 | Li et al. 2022 |
| *Arthrographis longispora* | CBS 135935 T | HG004555 | HG004540 | HG316559 | Li et al. 2022 |
| *Arthrographis longispora* | CBS 145528 | MN794372 | MN794349 | MN816510 | Li et al. 2022 |
| *Arthrographis multiformispora* | CGMCC 3.20770 = GZUIFR 21.926 T | OL475525 | OL475531 | OL589245 | Li et al. 2022 |
| *Arthrographis multiformispora* | GZUIFR 21.927 | OL475526 | OL475532 | OL589246 | Li et al. 2022 |
| *Arthrographis multiformispora* | GZUIFR 21.928 | OL475527 | OL475533 | OL589247 | Li et al. 2022 |
| *Arthrographis multiformispora* | GZUIFR 21.929 | OL475528 | OL475534 | OL589248 | Li et al. 2022 |
| *Eremomyces bilateralis* | CBS781.70 T | HG004552 | HG004545 | HG316562 | Li et al. 2022 |
| *Rhexothecium globosum* | CBS955.73 T | MH860827 | MH872561 | – | Li et al. 2022 |

Note: T = ex-type; new isolates in this study are in bold; the hyphen “–” represents the absence of a GenBank record. ITS: internal transcribed spacer region and intervening 5.8S nrRNA; LSU: 28S large subunit; ACT: actin gene.
